# O25 Catastrophic antiphospholipid crisis triggered by anticoagulant switch

**DOI:** 10.1093/rap/rkab067.024

**Published:** 2021-10-19

**Authors:** Rajinder Singh Andev, Shirish Dubey, Alexander Brand, Joel David, Raashid Luqmani

**Affiliations:** 1 University of Oxford, Oxford, United Kingdom; 2 Oxford University Hospitals NHS Foundation Trust, Oxford, United Kingdom

## Abstract

**Case report - Introduction:**

The COVID-19 pandemic led to drastic changes for some patients on warfarin for venous thromboembolic (VTE) disease and atrial fibrillation. Warfarin monitoring necessitates frequent interaction with healthcare workers, which is sufficiently risky for COVID-19 transmission. As a result, selected patients were swapped over to novel oral anticoagulants (NOACs). Our patient was changed without investigating for antiphospholipid syndrome (APLS); it later transpired he was triple antibody positive. He presented in a crisis and we describe his narrative. Patients on warfarin due to presumed unprovoked venous thromboembolic disease should not be swapped to NOACs without completing, or checking, previous antiphospholipid antibody testing.

**Case report - Case description:**

A 73-year-old gentleman presented locally in August 2020 with erythema over the anterolateral surface of his left leg. He was initially treated with antibiotics for presumed cellulitis. Within a few days this lesion became necrotic and rapidly spread. At this point, he was transferred to a tertiary rheumatology centre. Within days to weeks, he developed several necrotic lesions affecting his trunk and limbs, with facial sparing noted. Approximately 30—35% of his whole-body surface became involved.

He soon developed an oxygen requirement, with CTPA demonstrating lymphocytic interstitial pneumonitis without evidence of pulmonary emboli (PE). Throughout his admission, he had several other pathologies such as hyponatraemia that required level 2 care and severe non-infectious diarrhoea.

Skin biopsy identified thrombotic vasculopathy. Serology confirmed triple positive antiphospholipid antibody status and a dsDNA titre of > 400 iU/mL. This was the first-time serology had been undertaken despite a history of three deep vein thrombosis (DVT) episodes and two PE incidents. He had no history of SLE symptoms.

His initial management for vasculitis secondary to APLS at the point of limited necrosis consisted of IV methylprednisolone followed by rituximab and PO prednisolone. While there was some delay in the progression of his disease, new areas of necrosis arose, leading to the patient receiving cyclophosphamide. Low molecular weight heparin was used for anticoagulation.

This gentleman later developed proteinuria and neurological symptoms, fulfilling the criteria for catastrophic antiphospholipid syndrome. He received plasma exchange, without an improvement. He developed complications from his disease and treatment, including poor wound healing. It became apparent his condition would not improve and active treatments were stopped. He passed away 6 weeks after initial presentation.

Prior to his admission to hospital, his warfarin was swapped to a NOAC. This is thought to have been the trigger behind catastrophic thrombosis.

**Case report - Discussion:**

After excluding other conditions such as necrotising fasciitis, this gentleman was rapidly started on IV methylprednisolone to halt any further progression. This is because glucocorticoids have the greatest evidence base for managing this poorly understood acute disease manifestation.

After this failed to manage his condition, he was given a further immunosuppressive agent in the form of rituximab. This was used after his serology confirmed triple antibody status. It was hoped this would stop any further immunological mediated disease progression. Oral prednisolone was started at 40 mg at this stage and kept under review with a tapering schedule.

Cyclophosphamide was given within a few days of rituximab, with hope of a quicker onset of action. A careful MDT decision was made on these drug choices, particularly regarding their combined use and appreciating their side effect profiles. Cyclophosphamide has evidence behind its use, especially for those with APLS associated with lupus.

While he did not develop any infections related to treatment, his condition progressed. Case reports suggest that plasma exchange can be useful in the management of catastrophic antiphospholipid syndrome, so the team recommended this. Consent at this stage became tricky due to his altered mental status, but it was felt he did demonstrate capacity for this specific decision.

As his condition did not improve after this level of immunosuppression, the team reached the decision that no other treatments would likely change the outcome. He remained on oral steroids for the remainder of his admission.

The other management facet of APLS crises pertains to anticoagulation. Low molecular weight heparin was recommended by the haematologists. His NOAC was stopped after the diagnosis was confirmed. Warfarin was restarted later in his admission given he had been well on this for years.

**Case report - Key learning points:**

This fascinating case exemplifies the importance of completing an antiphospholipid antibody screen for patients who present with unprovoked venous thromboembolic disease.

NOACs are commonly used anticoagulant medications. Several case reports have demonstrated that patients with antiphospholipid syndrome experience breakthrough thromboembolic events when treated with NOACs. The highest risk is associated with history of arterial thrombosis and those with triple positive antibody status.

Three clinical trials have either been completed or are in the process of investigating whether NOACs sufficiently prevent thromboembolic disease in these patients. The TRAPS study compared rivaroxaban to warfarin in those with triple antibody positive antiphospholipid syndrome. The study was terminated early given that higher adverse events were observed in the rivaroxaban arm (19%, n = 11/59) versus warfarinised patients (3%, n = 2/61).

The RAPS study found no difference in thromboembolic risk and results from the ASTRO-APS study looking into apixaban are awaited. There is insufficient evidence to suggest that NOACs prevent VTE in a similar fashion to warfarin, so many still advocate the use of warfarin.

The optimal immune management of this acute complication is not well elucidated, with a shortfall in mechanistic pathological understanding. The conference will generate discussion on this subject matter in detail.

During the COVID-19 pandemic, it has been observed for patients to change anticoagulation from warfarin to NOACs. Given NOACs do not require monitoring, this medication change reduces the number of interactions patients have with healthcare services. We postulate this change triggered the crisis in our patient, where we suggest continuation of warfarin would have been ideal. This is due to the history of several unprovoked thromboembolic events without a prior antiphospholipid screen being completed. Dissemination of learning points from this case are imperative to ensure decision-making encompasses patients who may have undiagnosed antiphospholipid syndrome.

